# The Emerging Role of the Phosphatidylinositol 3-Kinase/ Akt/Mammalian Target of Rapamycin Signaling Network in Cancer Stem Cell Biology 

**DOI:** 10.3390/cancers2031576

**Published:** 2010-08-18

**Authors:** Alberto M. Martelli, Camilla Evangelisti, Francesca Chiarini, Cecilia Grimaldi, James A. McCubrey

**Affiliations:** 1 Department of Human Anatomy, University of Bologna, via Irnerio 48, 40126 Bologna, Italy; E-Mails: camilla.evangelisti@gmail.com (C.E.); francesca.chiarini@gmail.com (F.C.); ccgrimaldi@gmail.com (C.G.);; 2 Department of Microbiology & Immunology, Brody School of Medicine, East Carolina University, 600 Moye Boulevard, NC 27834, USA; E-Mail: mccubreyj@ecu.edu (J.A.M.)

**Keywords:** cancer stem cells, leukemic stem cells, PI3K/Akt/mTOR, proliferation, differentiation, targeted therapy

## Abstract

The cancer stem cell theory entails the existence of a hierarchically organized, rare population of cells which are responsible for tumor initiation, self-renewal/maintenance, and mutation accumulation. The cancer stem cell proposition could explain the high frequency of cancer relapse and resistance to currently available therapies. The phosphatidylinositol 3-kinase (PI3K)/Akt/mammalian target of rapamycin (mTOR) signaling pathway regulates a wide array of physiological cell functions which include differentiation, proliferation, survival, metabolism, autophagy, and motility. Dysregulated PI3K/Akt/mTOR signaling has been documented in many types of neoplasias. It is now emerging that this signaling network plays a key role in cancer stem cell biology. Interestingly, cancer stem cells displayed preferential sensitivity to pathway inhibition when compared to healthy stem cells. This observation provides the proof-of-principle that functional differences in signaling pathways between neoplastic stem cells and healthy stem cells could be identified. In this review, we present the evidence which links the signals emanating from the PI3K/Akt/mTOR cascade with the functions of cancer stem cells, both in solid and hematological tumors. We then highlight how targeting PI3K/Akt/mTOR signaling with small molecules could improve cancer patient outcome.

## 1. Introduction

The cancer stem cell (CSC) concept states that cancer initiation and propagation are driven by small subpopulations of cells displaying stem cell properties, such as the capability of self-renewal, asymmetric cell division, and differentiation. CSCs can sometimes arise from a normal stem cell or not, can undergo epigenetic changes, and are capable of differentiating into a phenotypically different progeny that ultimately forms subsets of non-tumorigenic cancer cells which compose the bulk of cells in a given tumor. These progeny cells have only a limited capacity to divide and survive [1]. The existence of CSCs may explain the occurrence of drug resistance and relapses in many tumors. Indeed, conventional anti-tumor treatments, such as chemotherapy with different drugs, could act only on more mature cancer cells, whereas CSCs are intrinsicly resistant, owing, in part, to elevated expression of ATP-binding cassette (ABC) family membrane transporters [2], and to the fact that CSCs are thought to have a low proliferation rate [3,4]. Therefore, the CSC theory highlights the need to develop new therapeutic strategies for eliminating immature neoplastic stem cells. 

Tumor cells displaying stem cell-like properties can be isolated using a variety of cell surface antigens and flow cytometry. This has allowed the initial characterization of specific signaling pathways in these putative CSCs as compared to both the bulk of tumor cells and their normal counterpart [5]. The phosphatidylinositol 3-kinase (PI3K)/Akt/mammalian target of rapamycin (mTOR) signaling cascade plays key roles in widely divergent physiological processes, which include cell cycle progression, differentiation, survival, transcription, translation, endocytosis, motility, metabolism, and autophagy [6]. Moreover, a clear link between this pathway and cancer was established as early as the 1980s, and in recent years it has become apparent that this signal transduction network is one of the most frequently aberrantly regulated pathways in human tumors [6]. Therefore, therapeutic targeting of the PI3K/Akt/mTOR axis by means of small molecule inhibitors is being pursued as an option for innovative treatment of several types of cancers [7,8]. The PI3K/Akt/mTOR signaling pathways is rapidly emerging as a signaling network important for CSC survival. In this review, we summarize and discuss the links between PI3K/Akt/mTOR signaling and CSC biology. Particular emphasis is placed on the emerging evidence suggesting that CSCs are more sensitive to pathway inhibition than healthy stem cells and could be a preferential target for small inhibitory molecules. 

## 2. The PI3K/Akt/mTOR Signaling Cascade

### 2.1. PI3K

PI3K phosphorylates the 3-OH group of the inositol ring of three species of phosphatidylinositol (PtdIns) lipid substrates; namely, PtdIns, PtdIns-4-phosphate (PtdIns 4P) and PtdIns-4,5-bisphosphate (PtdIns 4,5P2). PI3K products act as second messengers and mediate reversible membrane localization of cytoplasmic proteins which display lipid-binding domains, including the pleckstrin homology (PH) domain, the phox homology (PX) domain, and the Fab 1, YOTB, Vac 1, EEA1 (FYVE) domain. PI3K signaling contributes to many processes, which include cell cycle progression, cell differentiation, survival, migration, and intracellular vesicular transport.

There are three different PI3K classes: I, II, and III. In mammals, class I PI3Ks are present in all cell types. Class I PI3K is divided further into A and B subtype. Class IA PI3Ks are dimers comprising a regulatory (p85α, p85β, p55α, p55γ, p50α) and a catalytic (p110α, p110β, p110δ) subunit. Class I PI3Ks act downstream of both tyrosine kinase receptors (TKRs) and G protein-coupled receptors (GPCRs). The single class IB PI3K comprises a p110γ catalytic subunit which binds one of two related regulatory subunits, p101, and p87. Class IB PI3Ks are activated downstream of GPCRs [9].

p110α and p110β PI3Ks are ubiquitously expressed in mammalian tissues/organs and play fundamental roles during cell growth and development. Therefore, their homozygous knockout is embryonic-lethal [10]. In contrast, p110γ and p110δ PI3Ks are highly enriched in white blood cells, so that their knockdowns impair immune responses [11]. 

Mammals have three class II PI3K isoforms: PI3K-C2α, PI3K-C2β, and PI3K-C2γ. PI3K-C2α and PI3K-C2β isozymes have a broad but not ubiquitous tissue distribution, whereas the expression pattern of PI3K-C2γ seems to be more restricted. Class II PI3Ks do not have regulatory subunits, and upstream signaling inputs might be relayed through their extended N- and C-termini. The importance of class II PI3Ks in cell signaling and biology, relative to that of class I PI3Ks, is not entirely clear at the moment [12]. Class II PI3Ks are predominantly associated with intracellular membranes, with low levels in the cytosol and in the nucleus. They could be involved in cell motility and exocytosis [13]. 

There is only one class III PI3K, referred to as vacuolar protein sorting (vps) 34, which was originally identified in *Saccharomyces cerevisiae*. It exists as a heterodimer bound to the vps15 regulatory subunit (formerly called p150 in mammals). All known biological functions of vps34 in mammals are related to the regulation of vesicle traffic, including endocytosis, phagocytosis, and autophagy [14,15]

### 2.2. Akt

Akt is a 57-kDa serine/threonine kinase which belongs to the protein kinase A/protein kinase G/protein kinase C (AGC) protein kinase family. Akt, also referred as to as protein kinase B (PKB), is the cellular homolog of the v-akt oncogene. The Akt family includes three highly conserved isoforms: Akt1/α, Akt2/β, and Akt3/γ. While Akt 1 and Akt2 are ubiquitously expressed, Akt3 displays a more restricted tissue distribution and is found abundantly in nervous tissue [16]. Akt isoforms share structural homology, including a PH domain, an ATP binding site, and two phosphorylation sites. Despite their high sequence homology, Akt isoforms exert non-redundant functions. Indeed, Akt1 null mice have overall growth retardation, whereas Akt2 null mice develop insulin-resistance and diabetes. In contrast, Akt3 null mice display reduced brain size [17].

**Figure 1:**
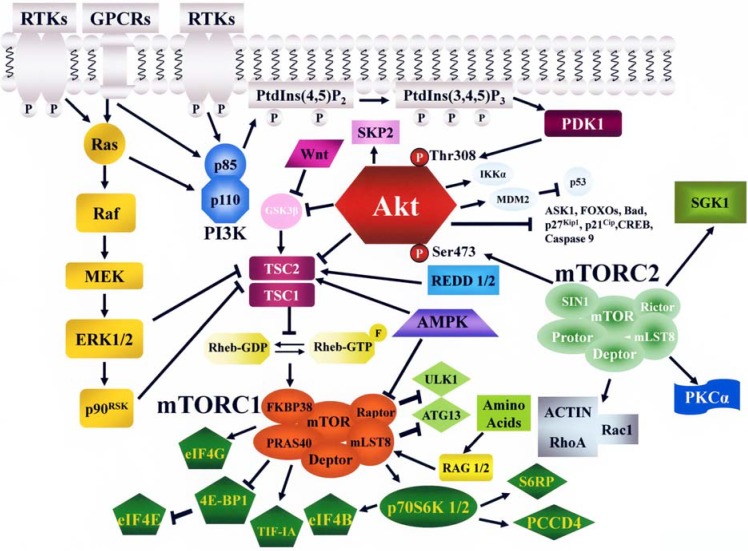
The PI3K/Akt/mTOR signaling pathway. RTKs, GPCRs, and Ras stimulate class I PI3K activity. PI3K generates PtdIns 3,4,5P3 from PtdIns 4,5, P2. PtdIns 3,4,5P3 attracts to the plasma membrane PDK1 which phosphorylates Akt on Thr 308. Full Akt activation requires Ser 473 phosphorylation by mTORC2. Active Akt inhibits TSC2 activity through direct phosphorylation. TSC2 is a GAP that functions in association with TSC1 to inactivate the small G protein Rheb. Akt-driven TSC1/TSC2 complex inactivation allows Rheb to accumulate in a GTP-bound state. Rheb-GTP upregulates the protein kinase activity of mTORC1. However, other signals impinge on mTORC1, including the Ras/Raf/MEK/ERK1/2/p90^RSK^ pathway, the AMPK network, RAG 1/2, REDD 1/2, Wnt/GSK3β. mTORC1 targets p70S6K 1/2 and 4E-BP1 which are critical for translation. S6RP, eIF4B, and PCCD4 are targets of p70S6K 1/2. mTORC2 regulates actin polymerization and phosphorylates PKCα and SGK1. Arrows indicate activating events, whereas perpendicular lines highlight inhibitory events.

The recruitment of Akt from the cytosol to the plasma membrane requires binding to PtdIns-3,4-bisphosphate (PtdIns 3,4P2) and PtdIns-3-4,5-trisphosphate (PtdIns 3,4,5P3) in the membrane through the PH domain within the *N*-terminus of Akt. Akt is then phosphorylated at Thr 308 within its catalytic domain by phosphatidylinositol-dependent kinase 1 (PDK1) and at Ser 473 within its *C*-terminus regulatory domain by mTOR complex 2 (mTORC2, see later on), resulting in full activation of Akt kinase (Figure 1). Active Akt migrates to the cytosol, the mitochondria, and the nucleus. Nuclear Akt fulfils important anti-apoptotic roles [18]. However, the relative contribution of Akt signaling at the various cellular domains remains to be established.

Around 40 substrates which mediate the pleiotropic Akt functions have been identified, including caspase-9, murine double minute 2 (MDM2, a negative regulator of p53), Bad, proline-rich Akt substrate 40 (PRAS40), Forkhead box class O (FOXO) family of transcription factors, IKβ kinase α (IKKα), apoptosis signal-regulated kinase 1 [ASK1, a negative regulator of pro-apoptotic c-Jun N-terminal kinase (JNK)], Raf, p27^Kip1^, p21^Cip1^, glycogen synthase kinase 3β (GSK3β), S-phase kinase-associated protein 2 (SKP2), and cAMP response element-binding protein (CREB). These substrates play key roles in the regulation of cell cycle progression, differentiation, and survival, either directly or through an intermediary [9,19,20]. 

### 2.3. mTOR

mTOR is the first identified member of the phosphatidylinositol 3-kinase-related kinase (PIKK) family, which includes Ataxia telangiectasia mutated (ATM); ATM and Rad3 related (ATR); DNA-dependent protein kinase (DNA-PK); suppressor with morphological effect on genitalia 1 (SMG1); and transformation/transcription domain-associated protein (TRRAP) [21]. Despite the homology to PI3K, mTOR has not been demonstrated to have lipid kinase activity, so that the significance of this homology remains unknown. mTOR was originally identified in the yeast *Saccharomyces Cerevisiae* as the target of rapamycin, a macrolide triene antibiotic with immunosuppressive properties produced by the bacterium *Streptomyces hygroscopicus*, which was isolated from a soil sample taken from Easter Island (known locally as Rapa Nui) [22]. 

**Figure 2:**
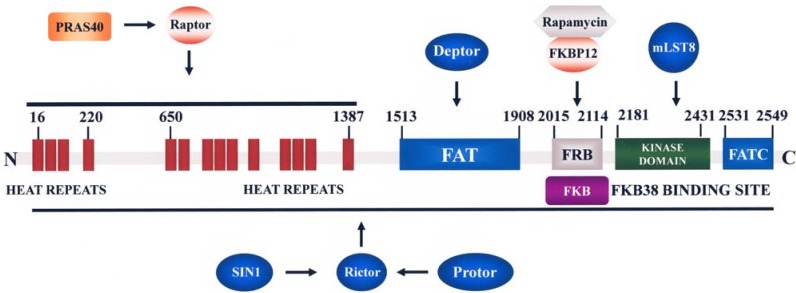
A schematic of mTOR structure. Some of the proteins interacting with mTOR domains are highlighted. The FRB domain is where the FKBP12 and rapamycin/rapalog complex binds, which is within the region that binds FKBP38.

The *N*-terminus of mTOR contains 20 tandem Huntingtin, Elongation factor 3, PR65/A subunit of protein phosphatase 2A, TOR (HEAT) repeats. This region is followed by a FRAP (FKBP12-rapamycin-associated protein/TOR), ATM, TRRAP (FAT) domain, an FK506-binding protein 12 (FKBP12) -rapamycin binding (FRB) domain, the kinase domain, and a C-terminus FATC domain. The HEAT repeats mediate protein-protein interactions, while the FAT and FATC domains which flank the catalytic site, modulate mTOR activity [23] (Figure 2). mTOR is a component of two notable multi-protein complexes, referred to as mTOR complex 1 (mTORC1) and mTORC2 (Figure 1). These complexes are characterized by their different partner proteins and their substrate specificity. mTORC1 is composed of mTOR/regulatory associated protein of mTOR (Raptor)/ mammalian Lethal-with-Sec-Thirteen 8 (mLST8, also referred to as GTPase β-subunit like, or GβL)/PRAS40/FKBP38 FK-506 binding protein 38 (FKBP38)/DEP-domain-containing mTOR interacting protein (Deptor), and is sensitive to rapamycin and its analogs (rapalogs) [22]. mTORC2 comprises mTOR/rapamycin-insensitive companion of mTOR (Rictor)/mLST8/stress-activated protein kinase-interacting protein 1 (SIN1)/ protein observed with Rictor (Protor)/Deptor, and is generally described as being insensitive to rapamycin/rapalogs. 

Rapamycin/rapalogs (RAD001, CCI-7709, AP23753) associate with FKBP12 and form complexes which bind the FRB domain of mTOR (Figure 2). This results in the dissociation of Raptor from mTORC1 and loss of contact between mTORC1 and its substrates.Therefore rapamycin/rapalogs act as allosteric mTORC1 inhibitors and do not directly affect the mTOR catalytic site [24,25].

However, there are mTORC1 functions, such as eukaryotic initiation factor 4E-binding protein 1 (4E-BP1) phosphorylation (see later on) which are not blocked by rapamycin/rapalogs [26,27]. Nevertheless, long-term (>24 hours) treatment of about 20% of cancer cell lines (mainly of hematopoietic origin) with rapamycin/rapalogs leads to dissociation of Rictor and SIN1 from mTORC2, resulting in its inhibition [28,29]. 

#### 2.3.1. Functions and Regulation of mTORC1

mTORC1 signaling integrates environmental clues (nutrients, oxygen levels, hormones, growth factors, stressors) and information from the cell metabolic status. Thus, mTORC1 controls anabolic processes to promote translation and cell growth [30]. mTORC1 regulates protein synthesis in response to nutrients/growth factors by phosphorylating p70S6 kinase (p70S6K) 1 and 2, and 4E-BP1. p70S6K 1/2 phosphorylates the 40S ribosomal protein, S6 (S6RP), leading to active translation of mRNAs. 

4E-BP1 phosphorylation by mTORC1 results in the release of the eukaryotic initiation factor 4E (eIF4E), which then associates with eukaryotic initiation factor 4G (eIF4G) to stimulate translation initiation. Thus, members of the 4E-BP family (4E-BP1, 4E-BP2, 4E-BP3) are suppressors of translation of a subset of transcripts referred to as “eIF4E-sensitive mRNAs” [31]. This mRNA subset is characterized by long and complex 5’UTR regions and encode proteins which are key regulators of cell proliferation and survival, including cyclins, c-Myc, Bcl-XL, Mcl-1, signal activator and transducer of transcription 3 (STAT3). Efficient translation of these mRNAs is achieved only when the activity of eIF4F is increased, that is usually observed in neoplastic cells [32]. In contrast, mRNAs encoding housekeeping proteins (e.g. actin), bear short, unstructured 5’UTR regions, and their translation rate is maximal even in cells requiring low protein synthesis such as non-transformed cells [30]. 

mTORC1 also modulates protein synthesis indirectly through the activation of TIF-IA (transcription initiation factor-IA, a key factor that transduces extracellular signals to the RNA polymerase I transcription machinery, see [33]) and consequent stimulation of transcription of rRNA and ribosomal biogenesis [34], as well as through phosphorylation of eIF4G [35]. Moreover, mTORC1 phosphorylates eukaryotic initiation factor 4B (eIF4B) and programmed cell death 4 protein (PCCD4) [36] (Figure 1).

Besides protein synthesis, it is now beginning to emerge that mTORC1 positively control lipid synthesis through the action of transcription factors such as sterol response element binding protein 1 (SREBP1) and peroxisomal proliferator-activated receptor γ (PPARγ) which regulate lipid and cholesterol homeostasis [37]. Furthermore, mTORC1 inhibits autophagy, an evolutionary-conserved, starvation-induced catabolic process wherein cellular organelles and/or long-lived proteins are enclosed in a double membrane structure and delivered to lysosomes for degradation [38,39]. Repression of autophagy by mTORC1 is achieved through phosphorylation of two autophagy promoting factors, unc-51-like kinase 1 (ULK1) and autophagy-related gene 13 (ATG13) [40,41,42] (Figure 1).

Akt-mediated regulation of mTORC1 activity involves several mechanisms. Akt phosphorylates Tuberous Sclerosis 2 (TSC2 or hamartin). TSC2 is a GTPase-activating protein (GAP) which associates with Tuberous Sclerosis 1 (TSC1 or tuberin) for inactivating the small G protein Ras homolog enriched in brain (Rheb). Once phosphorylated by Akt, TSC2 binds 14-3-3 protein and this reduces the TSC1/TSC2 complex GAP activity, allowing Rheb to accumulate in a GTP-bound state. The mechanism by which Rheb-GTP activates mTORC1 has not been fully elucidated yet, although Rheb requires farnesylation for activating mTORC1 [43]. Akt also phosphorylates PRAS40, an inhibitor of the interactions between mTORC1 and its substrates, and by doing so, Akt prevents PRAS40’s ability to suppress mTORC1 signaling [44]. Moreover, PRAS40 is a substrate of mTORC1 itself, and it has been demonstrated that mTORC1-mediated phosphorylation of PRAS40 relieves its inhibition on mTORC1 [45]. Nevertheless, the Ras/Raf/mitogen-activated protein kinase kinase (MEK)/extracellular signal-regulated kinase (ERK) 1/2 signaling network positively regulates mTORC1 activity, as both ERK 1/2 and p90 ribosomal S6 kinase (p90^RSK^) phosphorylate TSC2, thus suppressing its inhibitory function on Rheb [46]. Another signaling pathway which impinges on mTORC1 is the Wnt/GSK3β cascade [47], as it has been documented that Wnt stimulation could activate mTORC1 by inhibiting the TSC2 phosphorylation driven by GSK3β [48] (Figure 1). mTORC1 signal transduction is inhibited by the master metabolic regulator, energy-sensing AMP-dependent protein kinase (AMPK). Low energy levels (high AMP:ATP ratio) activate AMPK which then phosphorylates TSC2 on Ser 1345. This phosphorylation primes TSC2 for subsequent phosphorylation by GSK3β on Ser 1337 and Ser 1341 [49]. Indeed, the coordinated phosphorylation of TSC2 by AMPK and GSK3β is required for maximal activation of TSC2 and inhibition of mTORC1 [21,48]. However, AMPK also phosphorylates Raptor and this phosphorylation induces 14-3-3 protein binding to Raptor. The phosphorylation of Raptor by AMPK is necessary for the inhibition of mTORC1 and cell-cycle arrest induced by energy stress [50]. Mutations of the AMPK upstream activating kinase, LKB1 (also referred to as serine/threonine-protein kinase 11, or STK11), result in hyperactive mTORC1 signaling, thereby linking LKB1 to the TSC1/2 mTORC1 pathway.

Cellular stresses, such as hypoxia, inhibit mTORC1 signaling, through the HIF1-mediated upregulation of two homologous proteins, Regulated in Development and DNA damage response genes 1 and 2 (REDD 1 and REDD 2) [51,52,53]. REDD 1/2 act to activate TSC1/2, independently of LKB1-AMPK, in order to inhibit mTORC1. The stress and energy signaling pathways are likely to be associated, as prolonged hypoxia leads to ATP depletion and activation of AMPK [54]. Finally, amino acid uptake controls mTORC1 through the small GTPases of the Ras superfamily, Ras-related GTPase (RAG) 1 and 2 (Figure 1). RAG proteins target mTORC1 to a Rab7-positive late endosomal compartment that contains Rheb-GTP [55,56].

#### 2.3.2. Functions and Regulation of mTORC2

mTORC2 phosphorylates Akt on Ser 473 that enhances subsequent Akt phosphorylation on Thr 308 by PDK1 [57]. The oncogenetic role of mTORC2 has been recently highlighted by an investigation that documented the importance of mTORC2 in the development and progression of prostate cancers induced in mice by phosphatase and tensin homolog deleted on chromosome 10 (PTEN) loss [58]. Another downstream target of mTORC2 is the Ser 422 residue of serum- and glucocorticoid-induced protein kinase 1 (SGK1) [59]. There is now evidence that mTORC2 could facilitate cell proliferation through SGK1 and not Akt, at least in some experimental models [20]. Moreover, mTORC2 plays a role in cytoskeleton organization by regulating actin polymerization [60], phosphorylation of protein kinase C (PKC) α [61], and activation of Ras homolog gene family, member A (RhoA) and Ras-related C3 botulinum toxin substrate 1 (Rac1) [62]. The mechanisms which control mTORC2 activity have only begun to be revealed. mTORC2 activation requires PI3K and the TSC1/TSC2 complex, but is independent of Rheb and is largely insensitive to either nutrients or energy conditions [63]. 

#### 2.3.3. Feedback Loops Linking mTORC1/2 with Akt and MEK/ERK Signaling

mTORC1/2 and Akt are linked to each other through negative and positive regulatory feedback loops. These loops restrain simultaneous hyperactivation of mTORC1/2 and Akt through mechanisms involving p70S6K and PI3K. Once mTORC1 is activated through Akt, the former elicits a negative feedback circuit that inhibits Akt activity [28]. This negative regulation of Akt activity by mTORC1 is dependent on p70S6K-mediated phosphorylation of insulin receptor substrate (IRS) 1 adapter protein, downstream of insulin receptor and/or insulin-like growth factor-1 receptor [64,65,66], as IRS1 phosphorylation on Ser 307 and Ser 636/639 by p70S6K targets the adapter protein to proteosomal degradation [67]. A similar negative feedback loop controls the activity of MEK/ERK [68]. mTORC1 is capable of downregulating also IRS2 expression by enhancing its proteosomal degradation [69]. Recent findings have also highlighted the existence of a rapamycin-sensitive, mTORC1/p70S6K-mediated phosphorylation of Rictor on Thr 1135. This phosphorylative event exerted a negative regulatory effect on the mTORC2-dependent phosphorylation of Akt *in vivo* [70]. Thus, both mTORC1 and mTORC2 could control Akt activation. However, a more recent report could not confirm that Thr 1135 p-Rictor is important for regulating the levels of Ser 473 p-Akt [71].

At least in theory, the existence of these loops could limit the antitumor effects of rapamycin/rapalogs, and has prompted testing of drug combinations consisting of rapamycin/rapalogs and PI3K or MEK inhibitors [68,72].

## 3. Negative Regulation of PI3K/Akt/mTOR Signaling

The activity of several phosphatases is instrumental to counterbalance PI3K/Akt/mTOR signaling activation. PTEN is a dual specificity lipid and protein phosphatase that removes the 3’-phosphate mainly from PtdIns 3,4,5P3, but is also active on PtdIns 3,4P2, thereby antagonizing network signaling [73,74]. PTEN loss of function is associated with inherited cancer predisposition syndromes (for example, Cowden’s disease) and also occurs in a wide variety of sporadic human cancers [gliomas, melanomas, prostate, lung, renal, endometrial, breast and ovarian cancers, as well as T-cell acute lymphoblastic leukemia (T-ALL)] displaying Akt/mTOR up-regulation. Two other phosphatases, Src homology domain-containing inositol phosphatase (SHIP) 1 and 2, remove the 5-phosphate from PtdIns 3,4,5P3 to yield PtdIns 3,4P2 [75]. SHIP1 is a hematopoietic specific inhibitory molecule whose alterations could contribute to the development of certain types of malignant hematopoietic disorders, including chronic myelogenous leukemia (CML), acute myelogenous leukemia (AML), and T-ALL [76]. Protein phosphatase 2A (PP2A) downregulates Akt activity directly, by dephosphorylating Thr 308 and accumulating evidence indicates that PP2A acts as a tumor suppressor [77]. Additionally, Thr 308 and Ser 473 residues of Akt are targeted by the two isoforms (1 and 2) of PH domain leucine-rich repeat protein phosphatase (PHLPP). Decreased PHLPP activity has been linked to certain kinds of cancers, including breast and colon carcinomas, glioblastomas, and CML [78,79].

## 4. PI3K/Akt/mTOR Signaling in Leukemic Stem Cells (LSCs)

It is generally thought that leukemogenesis involves a series of alterations, that ultimately transform a healthy hematopoietic stem cell (HSC) or a committed hematopoietic progenitor cell, into a LSC capable of propagating the disease [80]. LSCs share some properties with HSCs, as they are for the most part quiescent and capable of self-renewing. The quiescence of LSCs could explain the difficulties in eradicating this cell population by conventional polychemotherapy and the relapses which characterize, for example, AML [5].

Over the last five years, several manuscripts have focused on the effects of PI3K/Akt/mTORC1 signaling activation in HSCs in relationship with the development of malignant hematological disorders, including leukemias. Murine HSCs without functional PTEN, started to move out of the bone marrow, colonized distant organs, and resulted first in a myeloproliferative disorder (MPD) and then an acute myeloid/lymphoid leukemic-like disease [81,82]. Rapamycin prevented the development of leukemia, implying an important role for mTORC1 in leukemogenesis. In another recent study, a conditional PTEN knockout mice was crossed with a myeloid-specific Cre line in which the Cre recombinase gene was inserted into the endogenous M lysozyme locus, and therefore was under the control of myeloid-specific lysozyme promoter. As a result, only in the myeloid linage was there a disruption of PTEN. In mice older than three months, a leukemia (which resembled human monocytic leukemia) was observed in 11 of 18 cases examined [83]. Moreover, PTEN functions as a tumor suppressor also for CML LSCs [84]. Also SHIP1 could be involved in leukemogenesis, as in Friend Murine Leukemia Virus-infected, SHIP1 knockout mice, accelerated erythroleukemia progression has been reported [85].

As to other components of this signaling pathway, p85α PI3K was demonstrated to be involved in oncogenic c-Kit-induced transformation in AML and systemic mastocytosis, in a model where p85α PI3K expression was disrupted in HSCs and mast cell progenitors. In contrast, p85β PI3K disruption had no apparent functional consequences [86]. Moreover, a myristoylated (constitutively active) allele of Akt1 (myr-Akt) was introduced into murine HSCs via retroviral transduction. HSCs in the myr-Akt mice displayed transient expansion and increased cycling, which, however, were associated with impaired engraftment and subsequent depletion of the HSC pool [87]. Expression of myr-Akt was sufficient to induce by 6–8 weeks an MPD and a T-cell lymphoma with high frequency (90% and 65%, respectively), and an AML with a lower penetrance (10%, without any evidence of preexisting MPD). The importance of mTORC1 signaling in T-cell lymphoma (but not MPD or AML) pathogenesis was suggested by the significantly increased survival observed when myr-Akt mice were treated with rapamycin. Another mTORC1 regulator potentially involved in leukemogenesis is TSC1, as TSC1 knockout mice developed an MPD [88]. However, in another mouse model where TSC1 was deleted, no MPD was observed. Instead, the authors reported a reduction of myeloid development [89]. A possible explanation for these conflicting results is that different deletor mouse strains were used in these two studies [90]. However, it may also be that there are TSC1/mTORC1-independent mechanisms mediating PI3K/PTEN/Akt signaling functions in HSCs that could be involved in leukemogenesis. In any case, it is very important to emphasize here that mTORC1 upregulation exerted a potent pro-survival effect in human LSCs tranplanted in NOD/SCID mice [91]. This finding indicated that therapeutical targeting of mTORC1 has the potential for eradicating AML.

In contrast, no AML was observed in a conditional FOXO deletion model [92], despite strong similarities in the HSC phenotype with the myr-Akt mice or the conditional PTEN deletion model. Thus, this study would imply that FOXO transcription factors contribute to maintenance of normal HSC homeostasis, but are not involved in leukemogenesis. This suggests that alternative or additional downstream targets of Akt, such as mTORC1, are required for leukemic transformation. 

However, FOXO transcription factors have been shown to be essential for the maintenance of LSCs in a murine model of CML. In particular, TGF (transforming growth factor) β/Akt signaling controlled FOXO3a nuclear localization. Moreover, serial CML transplantation experiments documented that FOXO3a deficiency severely impaired the ability of LSCs to induce CML [93]. A combination strategy consisting of both TGFβ and Bcr-Abl kinase inhibition and FOXO3a deficiency resulted in efficient depletion of LSCs and suppressed CML development [93].

## 5. PI3K/Akt/mTOR Signaling in CSCs

As in LSCs, an important role for PTEN inactivation/deletion in conferring CSC properties has been reported in several types of solid tumors, which include glioblastoma [94], hepatocellular carcinoma (HCC) [95], prostate carcinoma [96,97], lung adenocarcinoma [98], and breast carcinoma [99]. Of note, in transformed human prostate epithelial cells lacking PTEN, mTORC2 activity was required to form tumors after injection in immunocompromised mice. However, mTORC2 activity was dispensable for the function of normal prostate epithelial cells [58]. 

Similar results have been reported when constitutively active Akt was overexpressed in prostate carcinoma [100,101]. Intriguingly, when PI3K activity was inhibited by LY294002 in a model of colon carcinoma, the predominant goblet cell-like differentiation program of CSCs shifted to a more enterocyte-like differentiation [102]. Thus, this finding supported the concept that tumor hierarchy could be traced back to a single CSC capable of multilineage differentiation, and provided clues to the regulation of differentiation in colon cancers *in vivo*. In a recent report, it was demonstrated that Akt1 (but not Akt2) deletion promoted TGFβ-induced epithelial-mesenchymal transition and a stem-cell like phenotype in the breast epithelial cell line, MCF10A. Interestingly, this was accompanied by a downregulation of miR-200 family which controls E-cadherin expression levels [103].

## 6. Therapeutic Targeting of PI3K/Akt/mTOR Signaling in LSCs and CSCs

It is well-established that upregulation of PI3K/Akt/mTOR signaling is important for conferring a growth advantage to tumor cells. Moreover, pathway activation negatively influences tumor response to conventional anti-neoplastic treatments, including chemotherapy and radiotherapy [104]. Although most of the investigations performed in this field presented results obtained with the bulk of neoplastic cells, emerging evidence indicates that small molecules targeting PI3K/Akt/mTOR could display efficacy also against LSCs and CSCs.

Rapamycin, in combination with etoposide, decreased the engraftment activity of human LSCs from AML patients [91], while a dual PI3K/mTOR inhibitor, PI-103, targeted the CD34^+^/CD38^−^/CD123^+^ AML subpopulation which is enriched in LSCs [105]. 

In CD133^+^ HCC CSCs, Akt inhibition by a Akt1-specific inhibitor lowered drug-resistance to both doxorubicin and fluouracil, possibly through a mechanism involving Bad phosphorylation and Bcl-2 expression [106]. 

However, another likely mechanism which could explain decreased drug-resistence in response to Akt inhibition, is related to the fact that Akt controls ABCG2 expression on the plasma membrane [107]. Intriguingly, doxorubicin is a substrate for ABCG2 [108], however, available evidence suggests that fluorouracil is not [109]. The so-called side-population (SP) is thought to be enriched in CSCs. SP cells actively extrude the nuclear acid-staing dye Hoechst 33342 owing to high expression on their plasma membrane of drug resistance transporters of the ABC family such as ABCB1 and ABCG2, and can be identified by flow cytometry [110,111]. The enrichment of SP in LSCs/CSCs has been demonstrated in T-ALL [112], AML [113], lung cancer [114], prostate cancer [115], breast cancer [116], glioblastoma [117], and HCC [118].

In the SPs of both mouse and human glioblastomas (that are enriched in CSCs), Akt inhibition by the synthetic alkylphosphocholine, perifosine, lowered ABCG2 expression on the plasma membrane and decreased efflux of mitoxantrone, another ABCG2 substrate [119]. A similar phenomenon has been reported in the SP of a HCC cell line, MHCC97L [120]. Akt inhibition by the small molecule A-443654 inhibited the growth of CSCs from glioblastoma cell lines [121].

Another important clue to the role played by PI3K/Akt/mTOR signaling in conferring CSC resistance to therapeutic treatments, comes from a study which has documented that in mice medulloblastoma CD133^+^/nestin^+^ CSCs (which are located in perivascular niches), radiation activated Akt/mTOR signaling. Irradiated cells became quiescent after 6 hour of irradiation, but then re-entered cell cycle after 72 hour [122]. At 6 hour after irradiation, CD133^+^/nestin^+^ CSCs residing in the perivascular niche displayed increased levels of Ser 473 p-Akt and of p-S6RP, which were indicative of PI3K/Akt/mTOR activation. Importantly, in mice treated with the Akt inhibitor, perifosine, for 3 days prior to irradiation, the radiation-elicited Akt activation was abrogated and enhanced levels of apoptosis were detected in the perivascular niche CSC population. Overall, these findings supported the concept that enhanced PI3K/Akt/mTOR signaling in medulloblastoma CSCs is responsible for radioresistence of these cells, and that Akt inhibition could increase radiosensitivity. 

## 7. Conclusions and Future Perspectives

The CSC hypothesis is generating a great deal of interest because of its potential clinical implications, as it indicates that the route for cancer eradication will require the use of strategies which expunge the root cause of the tumor. The findings reviewed in this article strongly suggest that increased PI3K/Akt/mTOR signaling activity is important for regulating some of the CSC properties, including resistance to both chemotherapy and radiotherapy. 

However, at least two fundamental issues remain open. First, would it be possible to specifically target PI3K/Akt/mTOR signaling in LSCs/CSCs, without affecting the functions of healthy stem cells? Indeed, evidence suggests that this pathway is important for the biology of normal stem cells, including HSCs [123]. However, there are preliminary data indicating that there exist subtle differences in how normal stem cells and LSCs/CSCs utilize the same signaling pathways. This has been demonstrated in LCSs treated with rapamycin [82], as well as in brain tumor or prostate carcinoma CSCs exposed to Akt inhibitors [97,124]. In these cases, the drugs affected LSCs/CSCs, but not healthy stem cells. These observations have provided the proof-of-principle that functional differences in signaling pathways between CSCs and normal stem cells could be identified and exploited for targeted therapy. 

Second, will inhibition of just one pathway be sufficient to eradicate LSCs/CSCs and consequently the tumor? Indeed, it is now clear that several other pathways are active in LSCs/CSC and are important for properties such as self-renewal, quiescence, and drug-resistance [125]. These include, for example, Notch signaling [126], Sonic Hedgehog signaling [127], and Wnt/β-catenin signaling [128]. Thus, it may be that an effective targeting of LSCs/CSCs will require the use of several inhibitors to shut down all of these pathways. In this connection, it has been recently documented that only a combined treatment consisiting of cyclopamine (a Sonic Hedgehog inhibitor) plus the mTORC1 inhibitor rapamycin and chemotherapy (gemcitabine) was capable of reducing the number of pancreatic carcinoma CSCs to virtually undetectable levels both *in vitro* and *in vivo* [129]. Neither cyclopamine or rapamycin alone or as supplements with chemotherapy were capable of effectively reducing the CSC pool, whereas the combination resulted in the complete loss of metastatic activity *in vivo*. At least in mice, the combined treatment was reasonably tolerated and translated into significantly long-term tumor-free survival. 

Ultimately, the most efficacious use of multiple inhibitors will be in the context of personalized medicine. Therefore, if LSCs/CSCs are to be successfully targeted in clinical settings, this will require a thorough understanding of how these signaling pathways function in both normal and malignant cells. 

Therefore, evaluation of cross-talks between signaling pathways aberrantly activated in LSCs/CSCs coupled to identification of specific PI3K/Akt/mTOR substrates and of their roles in the quiescence, proliferation, survival, and differentiation of LSCs/CSCs as compared to their normal counterpart, could provide the rationale for developing personalized pharmacological treatments aimed to tumor eradication. 
